# Which Age Matters? Comparing Chronological and Biological Age in Adolescent Adaptation to School-Based Physical Activity Interventions (Wrocław PEER-HEART Study)

**DOI:** 10.3390/children12121607

**Published:** 2025-11-26

**Authors:** Jarosław Domaradzki, Marek Popowczak, Katarzyna Kochan-Jacheć, Paweł Szkudlarek, Dawid Koźlenia, Eugenia Murawska-Ciałowicz

**Affiliations:** Faculty of Physical Education and Sport, Wroclaw University of Health and Sport Sciences, 51-612 Wroclaw, Poland; jaroslaw.domaradzki@awf.wroc.pl (J.D.); marek.popowczak@awf.wroc.pl (M.P.); katarzyna.kochan-jachec@awf.wroc.pl (K.K.-J.); pawelszkudlarek@op.pl (P.S.); eugenia.murawska-cialowicz@awf.wroc.pl (E.M.-C.)

**Keywords:** chronological age, biological maturity, maturity offset, high-intensity interval training (HIIT), adolescents, school-based intervention, VO_2_max, body fat percentage, blood pressure

## Abstract

**Highlights:**

**What are the main findings?**

• Chronological age (CA) was a stronger predictor of physiological adaptations to an eight-week HIIT program than biological maturity (MO).
• In girls, CA better explained changes in VO_2_max and body fat percentage, while in boys, the interaction between CA and MO predicted systolic blood pressure responses.

**What are the implications of the main findings?**

• Chronological age may better capture cumulative behavioral and environmental factors influencing adaptation to school-based HIIT.
• Physical education programs should consider age-sensitive and individualized approaches to optimize health-related outcomes in adolescents.

**Abstract:**

**Background/Objectives:** Relative age differences within the same school year may influence adolescents’ physiological adaptations to physical activity programs. While biological maturity (maturity offset, MO) is often considered a more relevant indicator than chronological age (CA), empirical evidence from school-based interventions remains limited. This study aimed to compare the predictive value of CA and MO in explaining health-related adaptations to an eight-week high-intensity interval training (HIIT) program delivered during physical education classes. **Methods:** A total of 256 adolescents (112 boys and 144 girls) participated in HIIT protocols integrated into regular lessons. Health-related outcomes included maximal oxygen uptake (VO_2_max), body fat percentage (BFP), systolic blood pressure (SBP), and diastolic blood pressure (DBP), assessed at baseline, post-intervention, and follow-up. Maturity offset (MO) was estimated using Moore’s method. Statistical analyses included MANOVA/ANOVA, linear regression, and dominance analysis, all stratified by sex. **Results:** Chronological age explained more variance in the studied outcomes than MO, particularly for BFP and VO_2_max among girls. In boys, a significant CA × MO interaction predicted SBP, indicating interdependence between both age indicators. Dominance analysis confirmed the overall predominance of CA as a predictor across most outcomes. **Conclusions:** Chronological age proved to be a stronger predictor of adaptation to school-based HIIT than biological maturity, suggesting that calendar age better reflects cumulative behavioral and environmental influences. These findings highlight the need for age-sensitive and personalized approaches when designing physical education interventions to optimize health-related outcomes in adolescents.

## 1. Introduction

In 2008, Poland introduced an education reform aimed at lowering the school-starting age, based on the assumption that children develop cognitive readiness earlier due to improved parental education and socialization [1]. From 2015 onwards, all six-year-olds were obliged to begin primary school, with some children entering as early as five years old if born late in the year [1]. As a result, secondary school cohorts now include students differing in chronological age (CA) by up to 1.5 years. Such variation may influence physical and cognitive development. Girls at this stage are often completing puberty, while boys are just beginning, creating heterogeneity in physical education (PE) classes. Although studies have examined the consequences of early school entry 1), evidence in the context of PE remains scarce.

Beyond CA, differences in biological age, typically assessed using maturity offset (MO), may further amplify disparities in physical performance. A critical “window of opportunity” for improvements in oxygen uptake, strength, and power has been identified around peak height velocity (PHV), usually spanning one to three years [2]. Thus, biological maturity is often considered a stronger predictor of adaptation to exercise than CA [3]. However, Ortega [4] reported discrepancies between CA and biological age in relation to cardiorespiratory performance and strength.

The benefits of incorporating high-intensity interval training (HIIT) into school PE are increasingly well-documented. Studies show positive effects on body fat percentage (BFP), cardiovascular parameters, and cardiorespiratory fitness (CRF) [5,6,7,8,9]. HIIT has also been shown to improve VO_2_max [6,10], though relatively few studies have evaluated its application in school-based PE. Notable exceptions include Yang [11], Liao et al. [12], and Koźlenia et al. [7], who reported improvements in adolescent body composition, fitness, and physical function. Other research confirms HIIT’s versatility across adolescence, with benefits spanning early to late stages [13,14,15,16].

Despite these promising outcomes, the interaction between biological maturity and responsiveness to HIIT remains underexplored. Some studies highlighted the role of biological age in strength, power, and speed [17], as well as in resistance training adaptations [18,19]. Yet empirical comparisons of CA and biological age as predictors of school-based HIIT outcomes are scarce.

Therefore, this study aimed to compare the predictive value of CA and biological age (MO) in explaining individual variability in health-related outcomes following a school-based HIIT intervention. Specifically, we examined: (1) the individual and combined predictive strength of CA and MO using linear regression models, and (2) their relative contributions through dominance analysis. We hypothesized that, due to substantial variation in CA within school classes, biological age would better explain intervention-related changes than CA.

## 2. Materials and Methods

### 2.1. The Ethics Approval

The study was approved by the Ethics Committee of the Wroclaw University of Health and Sport Sciences (ECUPE No. 33/2018; issued 31 October 2018). All procedures were conducted in accordance with the Declaration of Helsinki.

### 2.2. Trial Registration

The project was conducted in Wrocław, Poland (2024), as part of the state-funded program Science for Society II (project no. NdS-II/SP/0521/2023/01). It was prospectively registered at ClinicalTrials.gov (identifier: NCT06431230) under the acronym PEER-HEART (Physical Education dosE Response Health markErs Adolescents inteRval Training).

### 2.3. Participants

The a priori sample size for the parent school-based trial was determined using G*Power v3.1 for a repeated-measures MANOVA with eight groups (two schools × sex × experimental/control) and three measurements (baseline, post-intervention, follow-up). Assuming an effect size f = 0.20, α = 0.05, and power (1–β) = 0.95, the required minimum total sample size per age cohort was n = 310, allowing for ≈20% attrition [20]. This calculation guided recruitment and class allocation procedures for both 1st-year and 4th-year cohorts.

Recruitment took place in two high schools. Recruitment for the 1st-year cohort took place in the first week of February 2024, followed by pre-intervention measurements during the second week of February 2024. The 8-week intervention lasted until the first week of April 2024, and post-intervention measurements were completed in the second week of April 2024. Recruitment for the 4th-year cohort occurred in the first week of September 2024, with pre-intervention measurements in the second week of September 2024. The 8-week intervention lasted until the first week of November 2024, and post-intervention measurements were completed in the second week of November 2024.

**Figure 1:**
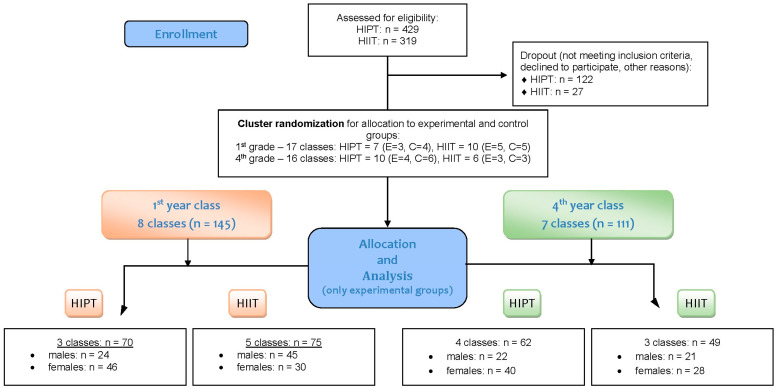
Flowchart depicting the process of participant inquiry (including the number of classes in the cluster randomization process).

Altogether, 429 first-year and 319 fourth-year students were assessed for eligibility. After exclusions due to refusal/consent issues, medical contraindications, additional sport participation, or >20% absence in PE lessons, 307 first-year and 292 fourth-year students remained in the study (organized into 17 classes in 1st year: HIPT = 7, HIIT = 10; and 16 classes in 4th year: HIPT = 10, HIIT = 6). Class-level allocation was performed using simple, non-returnable randomization (randomization.com; accessed on 8 January 2024). The participants inquiry was presented at the Figure 1.

Inclusion criteria were: regular PE participation, absence of medical contraindications to high-intensity exercise, no external HIIT, and written informed consent (with parental/guardian consent for minors). Exclusions included 79 students at baseline (refusal, health issues, or additional sport involvement) and 43 during the intervention for exceeding 20% absence. No adverse events occurred.

The present paper focuses exclusively on the experimental arms to compare effects according to calendar age (CA) versus biological age (maturity offset, MO). The experimental analytic sample comprised n = 256 adolescents (boys = 112, girls = 144):

• 1st year n = 145 (HIPT n = 70 (boys = 24, girls = 46); HIIT n = 75 (boys = 45, girls = 30))
• 4th year n = 111 (HIPT n = 62 (boys = 22, girls = 40); HIIT n = 49 (boys = 21, girls = 28)).

Controls are reported for transparency but were not analyzed here (1st year: n = 162; 4th year: n = 181). No adverse events were observed. The a priori power calculation was based on the full experimental–control trial to ensure adequate class-level recruitment and group balance. The present study focuses exclusively on the experimental arms to address a specific mechanistic question regarding the comparability of intervention effects according to calendar age and biological age (maturity offset). Therefore, while the achieved sample size (n = 256) represents a subset of the total trial population, it remains sufficient for within-experimental analyses stratified by sex and year. Although the parent PEER-HEART project was a cluster-randomized controlled trial including experimental and control arms, the present analysis focuses exclusively on the experimental groups (HIIT and HIPT). This design allows for an in-depth exploration of individual-level predictors (chronological and biological age) of training response, whereas the comparative effects versus control have been reported previously [21].

### 2.4. Procedures

Assessments were conducted at baseline, post-intervention, and after an eight-week follow-up. All measurements were completed within a single day (8:00–13:00) in sports halls under controlled conditions. Students wore standard sports attire, with shoes removed for anthropometry. Height and body composition were assessed first, while blood pressure and the multi-stage fitness test (MSFT) were administered on a separate day. Testing procedures were explained in advance to students, parents, and teachers.

### 2.5. Measurements

#### 2.5.1. Body Morphology

Standing height was measured to the nearest 0.1 cm using a GPM anthropometer (DKSH Ltd., Zurich, Switzerland) in accordance with ISAK protocols [22]. Body mass and body fat percentage (BFP) were assessed using a Tanita Inner Scan V BC-601 analyzer (Tanita Co., Tokyo, Japan), previously validated [21]. Standardized pre-test procedures (hydration, meal timing, bladder voiding) were followed. Measurements were taken barefoot and in minimal, standardized sportswear, with participants positioned according to manufacturer guidelines. BMI was calculated as body mass (kg)/height^2^ (m^2^).

#### 2.5.2. Blood Pressure

Blood pressure was measured with an Omron BP710 device (Omron Healthcare, Hoffman Estates, IL, USA) following the protocol of Nasier et al. [23]. Appropriate cuff sizes were selected based on arm circumference. After a 10 min seated rest, three measurements were taken at 10 min intervals; the mean value was used. At baseline, males presented mean SBP and DBP values of 125 ± 8.1 mmHg and 75 ± 6.4 mmHg, respectively, while females showed 120 ± 7.5 mmHg and 73 ± 6.2 mmHg, indicating a predominantly normotensive sample.

#### 2.5.3. Cardiorespiratory Fitness

Maximal heart rate (HRmax) was directly measured during the Multi-Stage Fitness Test (MSFT) using Polar Verity Sense sensors (Polar Electro, Kempele, Finland), while VO_2_max was subsequently estimated from test performance [24]. Participants ran 20 m shuttles at increasing pace (starting 8.5 km/h, increments of 0.5 km/h per minute) until exhaustion.

VO_2_max was estimated using the equation proposed by Ramsbottom et al. [25]:

VO_2_max = 3.46 × (L + SN/(L × 0.4325 + 7.0048)) + 12.2,

where L represents the final level completed and SN denotes the number of shuttles performed at that level.

### 2.6. Intervention

An eight-week HIIT program, based on the Tabata method, was implemented during regular PE lessons, with two sessions per week. Exercise intensity was individually prescribed as a percentage of maximal heart rate (HRmax), determined via the MSFT [26] and monitored using Polar Verity Sense sensors (Polar Electro, Kempele, Finland).

Each session began with a 10 min standardized warm-up. Training volume progressed incrementally: four work–rest rounds (20 s effort/10 s rest) in weeks 1–2, six rounds in weeks 3–4, and eight rounds in weeks 5–8. Two HIIT formats were applied:

HIPT variant: functional, whole-body movements (e.g., ankle hops, burpees, high knees, squat jumps, mountain climbers).

Classical HIIT variant: calisthenics (e.g., squats, lunges, push-ups, sit-ups, standing twists).

All sessions were supervised by trained instructors who provided feedback and ensured maximum effort.

During non-intervention PE classes, students followed the standard first-year curriculum (fundamental motor skills and sport-specific technique). Control groups completed only this curriculum, without HIIT exposure.

Only experimental groups were included in the present analysis. Session HR and intensity values confirmed vigorous training loads (grand means: ~77–80% HRmax across sex and year; detailed values in Supplementary Material).

The average HR and intensity across all sessions fell within the following range (grand mean–pooled values):

1st-year M: HR_average_–149.9 ± 3.75 (147.9–151.9); Intensity–0.80 ± 0.02 (0.78–0.82).

4th-year M: HR_average_–143.62 ± 8.29 (139.2–148.0); Intensity–0.77± 0.05 (0.74–0.80).

1st-year F: HR_average_–149.08 ± 2.7 (147.6–150.5); Intensity–0.79 ± 0.02 (0.78–0.80).

4th-year F: HR_average_–143.91 ± 5.87 (140.8–147.0); Intensity–0.77 ± 0.04 (0.75–0.80).

### 2.7. Biological Age Assessment

Biological maturity was estimated using sex-specific equations proposed by Moore et al. [27]. For each participant, maturity offset (MO, years from peak height velocity [PHV]) was calculated as:

Females: MO = 7.709133 + (0.0042232 × age × height)

Males: MO = 7.999994 + (0.0036124 × age × height)

Age at PHV (APHV) was then derived as calendar age minus MO. Participants were classified as biologically younger adolescents (BYA, lower MO, later maturation) or biologically advanced adolescents (BAA, higher MO, earlier maturation).

### 2.8. Statistics

Intervention effects were expressed as pre-to-post changes (Δ scores). Detailed baseline and post-intervention values are reported elsewhere [19]. Assumptions were checked using the Shapiro–Wilk test (normality) and Levene’s test (homogeneity of variances). Data are presented as mean ± SD with 95% CI.

Group-level differences were first examined using MANOVA (sex × training modality × school year), followed by univariate ANOVA. These analyses identified potential sources of variance to be considered in predictive modeling. To verify the independence assumption, intraclass correlation coefficients (ICCs) were computed for ΔBFP, ΔSBP, ΔDBP, and ΔVO_2_max, yielding values between 0.000 and 0.013 for the full sample. When examined separately by sex, the highest ICC (0.019 for ΔSBP in females) remained negligible. Given the low ICCs and the small number of clusters, mixed models with random class effects were deemed unnecessary, and the applied MANOVA/ANOVA and regression procedures were considered appropriate.

The primary analyses assessed the predictive value of chronological age (CA) and maturity offset (MO) for ΔBFP, ΔSBP, ΔDBP, and ΔVO_2_max. Sex was used as an a priori stratification factor, and training modality (HIIT vs. HIPT) was included only if significant in MANOVA/ANOVA. Simple linear regressions were run separately for CA and MO, followed by multiple regression models including both predictors and their interaction (CA × MO). For each model, R^2^, Akaike information criterion (AIC), and root mean square error (RMSE) were calculated to evaluate explanatory power, model fit, and prediction error.

Finally, dominance analysis was applied to quantify the relative contribution of CA and MO across all subset models, providing a robust ranking of predictor importance.

All analyses were performed in TIBCO Statistica v.13.3 (StatSoft, Kraków, Poland, 2018) and RStudio (Posit Software, Boston, MA, USA; accessed 15 May 2025) [28]. Statistical significance was set at α = 0.05.

## 3. Results

Descriptive statistics for chronological age (CA) and maturity offset (MO) are shown separately for sex and year in Table 1. In the 1st year, boys were 14.97 years old (SD = 0.52) with an MO of 1.75 years from PHV, while girls were 14.96 years (SD = 0.49) with an MO of 3.16 years. In the 4th year, boys were 18.36 years (SD = 0.49; MO = 3.95), and girls 18.23 years (SD = 0.50; MO = 5.49). As expected, girls were consistently more biologically advanced than boys of the same CA, with sex differences in MO of 1.42 years in the 1st year and 1.54 years in the 4th year.

Table 2 presents the baseline anthropometric and physiological characteristics of participants, including BMI, SBP, DBP, and VO_2_max. These data illustrate expected age-related differences between 1st- and 4th-year students and provide context for subsequent analyses examining the predictive roles of chronological and biological age. Between-sex statistical comparisons were not performed, as this was outside the study aims.

Table 3 presents the general pre–post intervention changes in body fat percentage, systolic and diastolic blood pressure, and maximal oxygen uptake by sex and year.

A three-way MANOVA revealed significant main effects of sex (Wilks’ Λ = 0.926, F = 5.01, *p* = 0.0007, partial η^2^ = 0.074) and year (Wilks’ Λ = 0.901, F(4,xx) = 6.87, *p* < 0.001, partial η^2^ = 0.099), but no significant sex × year interaction (Wilks’ Λ = 0.988, F = 0.76, *p* = 0.555, partial η^2^ = 0.012).

Univariate ANOVAs indicated that changes in body fat percentage (ΔBFP) were significantly affected by year (F = 23.47, *p* < 0.001), whereas neither sex (F = 1.32, *p* = 0.25) nor the sex × year interaction (F = 2.42, *p* = 0.121) reached significance.

For systolic blood pressure (ΔSBP), no significant effects were observed for sex (F = 0.67, *p* = 0.41), year (F = 2.11, *p* = 0.15), or their interaction (F = 0.28, *p* = 0.59).

Baseline SBP and DBP values were within the normotensive range for both sexes (males: 125/75 mmHg; females: 120/73 mmHg), confirming that the observed post-intervention reductions occurred in otherwise healthy, normotensive adolescents. For diastolic blood pressure (ΔDBP), none of the factors were significant, including sex (F = 0.47, *p* = 0.49), year (F = 1.39, *p* = 0.24), and the interaction (F = 0.04, *p* = 0.84).

In contrast, for maximal oxygen uptake (ΔVO_2_max), significant main effects of sex (F = 18.01, *p* < 0.001) and year (F = 4.59, *p* = 0.033) were found, while the interaction was not significant (F = 0.41, *p* = 0.52).

In summary, year strongly predicted changes in body fat, while sex was the main determinant of VO_2_max improvement. No significant predictors emerged for blood pressure changes.

The main analyses examined the predictive value of chronological age (CA) and maturity offset (MO) for intervention outcomes. Initial Pearson correlations between age indicators and changes in outcomes were weak to modest (r = 0.04–0.29; all *p* > 0.05), which justified the use of regression models (Table 4).

**Figure 2:**
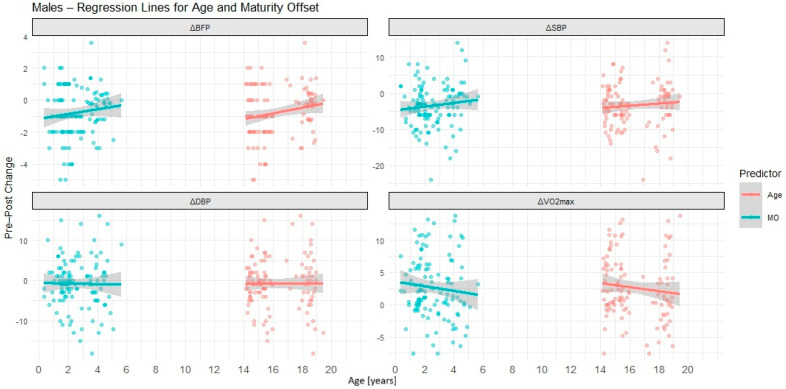
Simple linear regression lines with 95% confidence bands showing the relationships between chronological age (CA) and biological maturity offset (MO) and intervention-related changes in body fat percentage (ΔBFP), systolic blood pressure (ΔSBP), diastolic blood pressure (ΔDBP), and maximal oxygen uptake (ΔVO_2_max) in males. Each panel represents a different outcome variable. Predictor values (CA and MO, expressed in years) are displayed on a common *X*-axis for comparison purposes.

**Figure 3:**
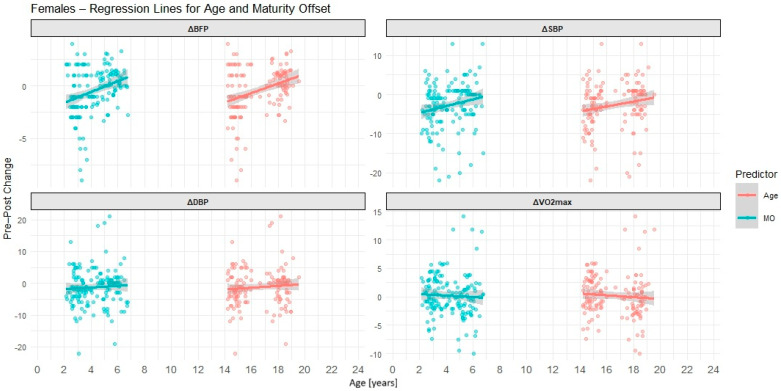
Simple linear regression lines with 95% confidence bands showing the relationships between chronological age (CA) and biological maturity offset (MO) and intervention-related changes in body fat percentage (ΔBFP), systolic blood pressure (ΔSBP), diastolic blood pressure (ΔDBP), and maximal oxygen uptake (ΔVO_2_max) in females. Each panel represents a different outcome variable. Predictor values (CA and MO, expressed in years) are displayed on a common *X*-axis for comparison purposes.

Table 5 presents simple linear regression models for chronological age and maturity offset as predictors of intervention outcomes based on sex. Graphically, results for males are presented in Figure 2, while results for females are showed in Figure 3.

In males, only CA significantly predicted changes in body fat percentage (ΔBFP), with older boys showing slightly smaller reductions (β = 0.18, *p* = 0.034). No other significant associations were observed, although coefficients for ΔSBP and ΔVO_2_max followed similar, non-significant patterns to those seen in females.

In females, both CA and MO were significant predictors of ΔBFP (CA: β = 0.45, *p* < 0.001; MO: β = 0.51, *p* < 0.001) and ΔSBP (CA: β = 0.63, *p* = 0.028; MO: β = 0.86, *p* = 0.022). These findings indicate that older and more biologically mature girls achieved smaller reductions in body fat and systolic blood pressure following the intervention. For ΔDBP and ΔVO_2_max, neither predictor was significant. However, the negative coefficients for ΔVO_2_max suggested a non-significant trend toward smaller aerobic gains with increasing age.

In males, the associations were weaker and limited mainly to body fat, suggesting that both chronological age (CA) and biological maturity (MO) exerted only a minor influence.

The multiple regression models included chronological age (CA), maturity offset (MO), and their interaction (Age × MO) to evaluate their combined predictive value for ΔBFP, ΔSBP, ΔDBP, and ΔVO_2_max, analyzed separately by sex.

In males, the simple model confirmed a significant effect of CA on ΔBFP (*p* = 0.034). However, in the multiple model, neither CA nor MO reached significance, although MO showed a trend toward significance (*p* = 0.096). For ΔSBP, the multiple regression model provided clearer evidence than the simple models: both CA (*p* = 0.029) and MO (*p* = 0.016) were significant predictors, and the interaction term (Age × MO) was also significant (*p* = 0.010). This indicates that the effect of one predictor depended on the level of the other, suggesting a synergistic relationship. No significant predictors were found for ΔDBP or ΔVO_2_max. Thus, in boys, multiple regression added explanatory value for systolic blood pressure by uncovering an interaction effect not visible in the simple models.

In females, the pattern was different. While simple regressions had shown significant associations of both CA and MO with ΔBFP and ΔSBP, the multiple regression models did not yield any significant predictors. All *p*-values exceeded 0.16, and the increase in standard errors suggested multicollinearity or shared variance between CA and MO. For ΔDBP and ΔVO_2_max, results were also non-significant, and the inclusion of the interaction term did not improve model fit (all *p* > 0.26).

The multiple regression models largely confirmed the simple regression findings for males, with the clearest result observed for ΔSBP, where a significant interaction between CA and MO was detected (Table 6). In contrast, for females, the simple models appeared more informative, as none of the predictors retained significance in the multiple model. These results indicate that the predictive value of CA and MO differs by sex and outcome, and that interaction effects may play an important role, particularly in relation to blood pressure responses among adolescent boys.

Summary of model fit statistics for simple and multiple regression models by sex and outcome variable are presented in Table 7. For body fat percentage (BFP) in males, the multiple model including age, MO, and their interaction achieved the best performance, with the highest explanatory power (R^2^ = 0.09), lowest AIC (416.36), and lowest RMSE (1.48). This suggests that combining chronological and biological age improved prediction compared to single-predictor models. For systolic blood pressure (SBP), all models showed low explanatory power (R^2^ ≤ 0.07), but the multiple model provided a slight improvement in fit, reducing AIC to 720.09 (vs. ~723) and RMSE to 5.76. In contrast, for diastolic blood pressure (DBP), R^2^ remained at 0.00 across all models, and AIC and RMSE values were nearly identical, indicating that none of the predictors meaningfully explained DBP changes.

Among females, the multiple model again provided the best fit for BFP (R^2^ = 0.13, RMSE = 2.08), although the model with age alone also performed well (R^2^ = 0.12). These results indicate that both age and MO accounted for meaningful variance in fat reduction among girls. For SBP, all models had low explanatory power (maximum R^2^ = 0.04). The model with MO alone yielded the lowest AIC (919.10), although predictive value remained limited. Similarly to males, none of the models explained a substantial portion of DBP variance (R^2^ ≤ 0.02), though the multiple model slightly reduced RMSE. For VO_2_max, predictive power was consistently poor across sexes and models (R^2^ ≤ 0.01), with minimal differences in AIC and RMSE.

Although the overall explained variance was low (R^2^ range = 0.00–0.13), the results consistently indicated that chronological age exerted a modest but statistically more stable influence than maturity offset. However, particularly among males, these effects should be interpreted with caution, as the low R^2^ values and inconsistent model fit indices (e.g., AIC and VO_2_max responses) suggest that the observed trends represent weak statistical tendencies rather than substantial physiological improvements.

Standardized β coefficients for all regression models are reported in Supplementary Table S1. These coefficients confirmed that chronological age exerted small-to-moderate effects (β ≈ 0.18–0.53), whereas maturity offset generally showed weaker associations. The observed predominance of CA over MO was therefore mainly statistical rather than practically substantial.

Dominance analysis was used to evaluate the relative contribution of chronological age (CA) and maturity offset (MO) to individual differences in intervention outcomes. This method compares predictors based on their incremental contribution to explained variance across all possible submodels.

In boys, the highest explained variance was observed for changes in body fat percentage (ΔBFP), with a total R^2^ of 0.077. On average, CA contributed 0.051 (~66% of the variance), while MO accounted for 0.025 (~34%). When added sequentially, CA increased R^2^ by 0.062 when added to a model already containing MO, whereas MO increased R^2^ by only 0.037 when added to CA. These results clearly identify CA as the dominant predictor of ΔBFP in males. For systolic blood pressure (ΔSBP), total explained variance was much lower (R^2^ = 0.012). Here, MO contributed slightly more on average (0.007) than CA (0.004), and adding MO second increased R^2^ by 0.004 compared with 0.001 for CA, suggesting a small relative advantage of MO in predicting ΔSBP. For diastolic blood pressure (ΔDBP), both predictors had negligible contributions (0.001 each), with equal ΔR^2^ values (0.002), indicating no meaningful difference. For maximal oxygen uptake (ΔVO_2_max), CA made a larger average contribution (0.009 vs. 0.005), and when added after MO, CA increased R^2^ by 0.005 compared with 0.001 for MO. Thus, for VO_2_max, CA provided more unique predictive information.

Table 8 presents the average coefficient of determination contribution of chronological age and maturity offset to explaining pre–post intervention changes in body fat percentage, systolic blood pressure, diastolic blood pressure, and maximal oxygen uptake.

In girls, the highest explained variance was again observed for body fat percentage (ΔBFP), with a total R^2^ of 0.123. Here, CA contributed more than MO (0.077 vs. 0.047). When added sequentially, CA increased R^2^ by 0.034 when added to MO, whereas MO increased R^2^ by only 0.004 when added to CA. This result reinforces the dominant role of CA in predicting fat changes among females.

For systolic blood pressure (ΔSBP), the average contributions of MO (0.019) and CA (0.017) were comparable. However, adding MO to CA improved R^2^ slightly (+0.003), whereas adding CA to MO did not increase explained variance at all, indicating a minimal advantage for MO. For diastolic blood pressure (ΔDBP), CA contributed more than MO (0.007 vs. 0.003), and when added sequentially, CA again provided a greater increase in R^2^ (0.007 vs. 0.003). Similarly, for maximal oxygen uptake (ΔVO_2_max), CA contributed more on average (0.007 vs. 0.003) and increased R^2^ more when added after MO (0.008 vs. 0.004).

Taken together, ΔBFP showed the highest explained variance in both sexes, with CA emerging as the dominant predictor. ΔSBP was the only outcome where MO showed a slight advantage, particularly among girls. For ΔDBP and ΔVO_2_max, total explained variance was low, but the overall pattern still favored CA.

## 4. Discussion

This study examined the relative importance of chronological age (CA) and biological age (maturity offset, MO) in predicting adolescent responses to a school-based HIIT program. Our analyses showed that CA was generally a stronger overall predictor of intervention outcomes than MO, particularly for body fat percentage (BFP), and a modest predictor for VO_2_max. An interaction between CA and MO was observed for systolic blood pressure (SBP) in boys, suggesting that both markers may jointly influence cardiovascular responses. These findings challenge the assumption that biological maturity would be the superior determinant of adaptation. While chronological age emerged as a statistically dominant predictor, the magnitude of explained variance was small, suggesting limited practical utility and supporting only a modest predominance of CA over MO.

The standardized effect sizes indicated that the impact of chronological age was small to moderate (β range ≈ 0.18–0.51), while that of maturity offset was generally weaker (β < 0.30). These findings confirm a modest statistical predominance of CA over MO but suggest limited practical relevance in explaining physiological variability.

The observed dominance of CA aligns with research indicating that calendar age may capture cumulative behavioral and environmental influences, such as training exposure, nutrition, and lifestyle habits [3,4]. Although girls were consistently more biologically advanced than boys of the same CA, CA explained more variance in their responses, particularly for body fat percentage (BFP), but not for systolic blood pressure (SBP). This partially contrasts with earlier studies suggesting stronger effects of maturation timing, for example, in physical fitness outcomes around peak height velocity [5,16]. However, our findings confirm that HIIT is effective in reducing adiposity and improving fitness in adolescents across a broad maturational range [6,7,8,9].

Because baseline SBP and DBP values were already within normal limits, the observed decreases should be interpreted as favorable cardiovascular adjustments rather than clinical normalization. These findings are consistent with previous evidence showing that high-intensity interval training can improve vascular compliance and autonomic balance even in normotensive youth.

Sex moderated the predictive value of age indicators. In girls, both CA and MO predicted outcomes, but CA consistently contributed more, suggesting that developmental advancement interacts with behavioral and environmental factors. In boys, associations were weaker and limited mainly to adiposity and SBP. These differences may reflect the earlier maturation of girls and the delayed but more pronounced growth spurts in boys [29,30]. The results highlight the importance of considering sex when interpreting the role of age in adolescent training responses.

For educators and practitioners, these findings suggest that while biological age offers valuable insights into maturation status, chronological age remains a pragmatic and often more powerful predictor in school settings. Simple age-based grouping may therefore be sufficient for structuring PE interventions, though caution is warranted when interpreting individual responses. Teachers and coaches should remain attentive to maturational differences, especially in strength and endurance tasks, to minimize injury risk and enhance engagement. More broadly, incorporating short bouts of HIIT into school curricula continues to represent a feasible and effective strategy for improving adolescent health and supporting Sustainable Development Goal 3 (good health and well-being).

This was an exploratory study and findings should not be interpreted as causal. Biological age was estimated using Moore’s method rather than skeletal or hormonal markers, which may limit precision. Effect sizes were modest, indicating that factors beyond age—such as motivation, baseline fitness, or family environment—also shape responses. Future research should incorporate more direct biological measures, longitudinal designs, and larger, more diverse samples. Investigating behavioral moderators such as diet, sleep, and stress could further clarify the interaction between maturational and environmental influences. Another limitation is that the analyses did not include random effects for class-level clustering. However, the ICCs for main outcomes were negligible (<0.02), indicating minimal dependency among students within classes. Therefore, the applied analytical approach is unlikely to have biased the estimates or inflated type I error.

## 5. Conclusions

Chronological age (CA) emerged as a more consistent and dominant predictor of adolescent responses to a school-based HIIT program than biological age (MO), particularly for changes in body fat and VO_2_max. Chronological age showed a modest statistical predominance over biological age, with limited explanatory power (R^2^ ≤ 0.13). Thus, while CA may better reflect cumulative behavioral and environmental influences, its predictive strength should be interpreted cautiously. This suggests that, despite expectations, calendar age may capture meaningful variation in physiological adaptation, possibly through accumulated lifestyle and environmental influences.

From a practical perspective, age heterogeneity within school classes should be considered when designing and evaluating PE programs. While the predictive value of age is modest, recognizing differences between students can support more individualized and effective approaches.

It should also be noted that the present work represents a secondary, hypothesis-driven analysis within the larger PEER-HEART randomized controlled trial. While the original study compared experimental and control groups, this analysis focused exclusively on the experimental arms to investigate mechanisms underlying individual variability in physiological adaptations.

Future studies should incorporate more precise biological markers and extended longitudinal designs with multiple follow-up assessments, as well as behavioral moderators such as physical activity, sleep, and diet, to better contextualize developmental readiness and clarify the respective roles of CA and MO over time.

## Figures and Tables

**Table 1:** Descriptive statistics for chronological age (CA) and maturity offset (MO) by sex and school year.

Sex	School Year	Mean CA (Years) ± SD	Mean MO (Years from PHV) ± SD
Boys	1st	14.97 ± 0.52	1.75 ± 0.60
Girls	1st	14.96 ± 0.49	3.16 ± 0.55
Boys	4th	18.36 ± 0.49	3.95 ± 0.64
Girls	4th	18.23 ± 0.50	5.49 ± 0.58

Footnote: SD—standard deviation.

**Table 2:** Baseline anthropometric and physiological characteristics of participants by sex and school year.

Variable	School Year	Males Mean ± SD	Females Mean ± SD
Body height (cm)	1st	175.82 ± 6.26	164.54 ± 5.86
Body weight (kg)		65.29 ± 10.71	56.65 ± 9.59
BMI (kg/m^2^)		21.08 ± 3.13	20.90 ± 3.21
SBP (mmHg)		125 ± 8.1	120 ± 7.5
DBP (mmHg)		75 ± 6.4	73 ± 6.2
VO_2_max (mL·kg^−1^·min^−1^)		44.6 ± 5.3	40.8 ± 4.9
Body height (cm)	4th	180.09 ± 6.82	165.89 ± 5.99
Body weight (kg)		72.12 ± 10.40	57.86 ± 9.02
BMI (kg/m^2^)		22.19 ± 2.55	20.97 ± 2.74
SBP (mmHg)		126 ± 8.4	121 ± 7.6
DBP (mmHg)		76 ± 6.5	74 ± 6.1
VO_2_max (mL·kg^−1^·min^−1^)		45.3 ± 5.0	41.2 ± 4.6

Footnote: BMI—body mass index; DBP—diastolic blood pressure; SBP—systolic blood pressure; VO_2_max—maximal oxygen uptake.

**Table 3:** Descriptive statistics for pre–post intervention changes in body fat percentage, systolic blood pressure, diastolic blood pressure, and maximal oxygen uptake by sex and year.

Variable	Year			Males			Females		
		Mean	Lower 95% CI	Upper 95% CI	SD	Mean	Lower 95% CI	Upper 95% CI	SD
∆BFP	1st	−1.09	−1.48	−0.69	1.64	−1.18	−1.77	−0.60	2.57
∆SBP		−3.65	−4.88	−2.43	5.09	−3.43	−4.72	−2.15	5.64
∆DBP		−1.06	−2.39	0.27	5.54	−1.75	−2.96	−0.54	5.28
∆VO_2_max		3.23	2.16	4.30	4.46	0.57	−0.11	1.25	2.99
∆BFP	4th	−0.30	−0.70	0.10	1.31	0.35	0.01	0.69	1.41
∆SBP		−2.95	−5.19	−0.72	7.27	−1.93	−3.40	−0.45	6.10
∆DBP		−0.30	−2.54	1.94	7.28	−0.68	−2.26	0.90	6.53
∆VO_2_max		1.71	0.07	3.36	5.36	−0.25	−1.32	0.83	4.44

Footnote: CI—confidence interval; ∆BFP—change in body fat percentage; ∆DBP—change in diastolic blood pressure; ∆SBP—change in systolic blood pressure; ∆VO_2_max—change in maximal oxygen uptake.

**Table 4:** Pearson correlation coefficients between chronological age (CA), maturity offset (MO), and pre–post intervention changes in body fat percentage (ΔBFP), systolic blood pressure (ΔSBP), diastolic blood pressure (ΔDBP), and maximal oxygen uptake (ΔVO_2_max).

Predictor	ΔBFP	ΔSBP	ΔDBP	ΔVO_2_max
CA	0.29	0.14	0.040	–0.108
MO	0.24	0.16	0.012	–0.205

Footnote: CA—chronological age, MO—maturity offset, Δ—delta (change), BFP—body fat percentage, SBP—systolic blood pressure, DBP—diastolic blood pressure, VO_2_max—oxygen uptake.

**Table 5:** Simple linear regression models for chronological age and maturity offset as predictors of intervention outcomes based on sex.

Sex	Variable	Term	Estimate	Std. Error	t-Value	*p*-Value
Male	ΔBFP	Age	0.18	0.08	2.14	**0.034**
		MO	0.15	0.12	1.26	0.211
	ΔSBP	Age	0.31	0.33	0.95	0.344
		MO	0.51	0.46	1.11	0.269
	ΔDBP	Age	0.01	0.34	0.02	0.981
		MO	−0.07	0.48	−0.15	0.884
	ΔVO_2_max	Age	−0.31	0.27	−1.18	0.239
		MO	−0.36	0.37	−0.97	0.332
Female	ΔBFP	Age	0.45	0.10	4.38	**0.000**
		MO	0.51	0.14	3.74	**0.000**
	ΔSBP	Age	0.63	0.28	2.22	**0.028**
		MO	0.86	0.37	2.31	**0.022**
	ΔDBP	Age	0.28	0.29	0.98	0.328
		MO	0.25	0.38	0.67	0.504
	ΔVO_2_max	Age	−0.16	0.18	−0.88	0.379
		MO	−0.13	0.24	−0.55	0.585

Footnote: ∆BFP—change in body fat percentage; ∆DBP—change in diastolic blood pressure; ∆SBP—change in systolic blood pressure; ∆VO_2_max—change in maximal oxygen uptake. Significant values at *p* < 0.05 are highlighted in bold.

**Table 6:** Multiple linear regression for chronological age, maturity offset, and their interaction as predictors of intervention outcomes based on sex.

Sex	Outcome	Term	Estimate	Std. Error	t-Value	*p*-Value
Males	BFP	Age	0.25	0.38	0.66	0.512
		MO	−2.86	1.70	−1.68	0.096
		Age × MO	0.13	0.10	1.31	0.194
	SBP	Age	−3.23	1.46	−2.21	**0.029**
		MO	−16.13	6.61	−2.44	**0.016**
		Age × MO	1.03	0.39	2.61	**0.010**
	DBP	Age	0.19	1.58	0.12	0.902
		MO	−2.02	7.12	−0.28	0.777
		Age × MO	0.08	0.43	0.20	0.844
	VO_2_max	Age	−0.90	1.22	−0.74	0.463
		MO	−1.45	5.51	−0.26	0793
		Age × MO	0.11	0.33	0.34	0.735
Females	BFP	Age	0.06	0.62	0.10	0.921
		MO	−2.78	2.24	−1.25	0.215
		Age × MO	0.15	0.13	1.12	0.265
	SBP	Age	−0.05	1.72	−0.03	0.977
		MO	−0.14	6.21	−0.02	0.982
		Age × MO	0.05	0.36	0.13	0.895
	DBP	Age	2.43	1.74	1.40	0.165
		MO	5.79	6.28	0.92	0.358
		Age × MO	−0.39	0.37	−1.05	0.293
	VO_2_max	Age	−0.92	1.11	−0.83	0.409
		MO	−1.02	4.01	−0.25	0.800
		Age × MO	0.09	0.24	0.39	0.697

Footnote: BFP—body fat percentage; DBP—diastolic blood pressure; SBP—systolic blood pressure; VO_2_max—maximal oxygen uptake. Significant values at *p* < 0.05 are highlighted in bold.

**Table 7:** Summary of model fit statistics for simple and multiple regression models by sex and outcome variable.

Sex	Outcome	MOdel	R^2^	AIC	RMSE
Males	BFP	Age	0.04	418.47	1.53
		MO	0.01	421.45	1.55
		Age + MO + Age × MO	0.09	416.36	1.48
	SBP	Age	0.01	723.35	5.95
		MO	0.01	723.01	5.94
		Age + MO + Age × MO	0.07	720.09	5.76
	DBP	Age	0.00	733.23	6.22
		MO	0.00	733.21	6.22
		Age + MO + Age × MO	0.00	736.96	6.21
	VO_2_max	Age	0.01	675.60	4.81
		MO	0.01	676.06	4.82
		Age + MO + Age × MO	0.01	679.34	4.80
Females	BFP	Age	0.12	626.97	2.09
		MO	0.09	631.74	2.12
		Age + MO + Age × MO	0.13	628.98	2.08
	SBP	Age	0.03	919.48	5.77
		MO	0.04	919.10	5.76
		Age + MO + Age × MO	0.04	923.05	5.76
	DBP	Age	0.01	924.17	5.87
		MO	0.00	924.69	5.88
		Age + MO + Age × MO	0.02	926.55	5.83
	VO_2max_	Age	0.01	794.02	3.73
		MO	0.00	794.50	3.74
		Age + MO + Age × MO	0.01	797.25	3.72

Footnote: AIC—Akaike information criterion; BFP—body fat percentage; DBP—diastolic blood pressure; MO—maturity offset; RMSE—root mean square error; R^2^—coefficient of determination; SBP—systolic blood pressure; VO_2_max—maximal oxygen uptake.

**Table 8:** Dominance analysis results for males and females. The table presents the average coefficient of determination contribution of chronological age and maturity offset to explaining pre–post intervention changes in body fat percentage, systolic blood pressure, diastolic blood pressure, and maximal oxygen uptake. Also shown are the additional coefficient of determination gains when each predictor was added second to the model, and the total coefficient of determination of the full model with both predictors.

Sex	Outcome	Avg R^2^ (Age)	Avg R^2^ (MO)	ΔR^2^ When MO Added to Age	ΔR^2^ When Age Added to MO	Total R^2^ (Age + MO)
Males	ΔBFP	0.051	0.025	0.037	0.062	0.077
	ΔSBP	0.004	0.007	0.004	0.001	0.012
	ΔDBP	0.001	0.001	0.002	0.002	0.002
	ΔVO_2_max	0.009	0.005	0.001	0.005	0.014
Females	ΔBFP	0.077	0.047	0.004	0.034	0.123
	ΔSBP	0.017	0.019	0.003	0.000	0.036
	ΔDBP	0.007	0.003	0.003	0.007	0.010
	ΔVO_2_max	0.007	0.003	0.004	0.008	0.010

Footnote: ΔBFP—change in body fat percentage; ΔDBP—change in diastolic blood pressure; MO—maturity offset; R^2^—coefficient of determination; ΔSBP—change in systolic blood pressure; ΔVO_2_max—change in maximal oxygen uptake.

## Data Availability

The raw data supporting the conclusions of this article will be madeavailable by the authors on request.

## Supplementary Materials

The following supporting information can be downloaded at: https://www.mdpi.com/article/10.3390/children12121607/s1, Supplementary Table S1: Standardized regression coefficients (β) with 95% confidence intervals for all models (chronological age, biological maturity, and their interaction) across outcomes (ΔBFP, ΔSBP, ΔDBP, and ΔVO_2_max) and sex. The table presents the standardized effect sizes derived from z-scored variables, allowing direct comparison of predictor magnitudes. These data support the main findings by confirming that chronological age exerts a modest but statistically predominant influence over maturity offset, with limited explanatory power.
