# Supplementary material for: Which Age Matters? Comparing Chronological and Biological Age in Adolescent Adaptation to School-Based Physical Activity Interventions (Wrocław PEER-HEART Study)
Source: Children (Basel). 2025 Nov 26;12(12):1607. doi: 10.3390/children12121607 (PMC12732137; doi:10.3390/children12121607)
Supplement: Supplementary file 1 [file children-12-01607-s001.zip › children-3946130-supplementary.pdf]

Supplementary Table S1. Standardized β coefficients with 95% confidence intervals for regression models by sex and outcome.

Sex	Outcome	Model	Predictor	Beta_standardized	CI_lower	CI_upper	p_value
F	ΔBFP	age	Age	0.345	0.189	0.501	<0.001
F	ΔBFP	age_mo_int	Age	0.527	0.089	0.965	0.019
F	ΔBFP	age_mo_int	Age:MO	0.146	-0.112	0.405	0.265
F	ΔBFP	age_mo_int	MO	-0.209	-0.649	0.231	0.349
F	ΔBFP	mo	MO	0.299	0.141	0.457	<0.001
F	ΔDBP	age	Age	0.082	-0.083	0.247	0.328
F	ΔDBP	age_mo_int	Age	0.225	-0.241	0.691	0.341
F	ΔDBP	age_mo_int	Age:MO	-0.146	-0.421	0.128	0.293
F	ΔDBP	age_mo_int	MO	-0.138	-0.606	0.329	0.56
F	ΔDBP	mo	MO	0.056	-0.11	0.222	0.504
F	ΔSBP	age	Age	0.183	0.02	0.346	0.028
F	ΔSBP	age_mo_int	Age	0.046	-0.416	0.507	0.846
F	ΔSBP	age_mo_int	Age:MO	0.018	-0.254	0.29	0.895
F	ΔSBP	age_mo_int	MO	0.146	-0.318	0.609	0.535
F	ΔSBP	mo	MO	0.19	0.027	0.353	0.022
F	ΔVO2max	age	Age	-0.074	-0.239	0.092	0.379
F	ΔVO2max	age_mo_int	Age	-0.241	-0.709	0.226	0.309
F	ΔVO2max	age_mo_int	Age:MO	0.054	-0.221	0.33	0.697
F	ΔVO2max	age_mo_int	MO	0.174	-0.296	0.643	0.466
F	ΔVO2max	mo	MO	-0.046	-0.212	0.12	0.585
M	ΔBFP	age	Age	0.2	0.015	0.385	0.034
M	ΔBFP	age_mo_int	Age	0.656	0.139	1.173	0.013
M	ΔBFP	age_mo_int	Age:MO	0.182	-0.094	0.457	0.194
M	ΔBFP	age_mo_int	MO	-0.556	-1.069	-0.044	0.034
M	ΔBFP	mo	MO	0.119	-0.069	0.307	0.211
M	ΔDBP	age	Age	0.002	-0.187	0.191	0.981
M	ΔDBP	age_mo_int	Age	0.114	-0.427	0.655	0.677
M	ΔDBP	age_mo_int	Age:MO	0.029	-0.26	0.318	0.844
M	ΔDBP	age_mo_int	MO	-0.13	-0.667	0.407	0.631
M	ΔDBP	mo	MO	-0.014	-0.203	0.175	0.884
M	ΔSBP	age	Age	0.09	-0.098	0.278	0.344
M	ΔSBP	age_mo_int	Age	-0.161	-0.684	0.361	0.542
M	ΔSBP	age_mo_int	Age:MO	0.367	0.089	0.646	0.010
M	ΔSBP	age_mo_int	MO	0.131	-0.387	0.65	0.616
M	ΔSBP	mo	MO	0.105	-0.082	0.293	0.269
M	ΔVO2max	age	Age	-0.112	-0.3	0.076	0.239
M	ΔVO2max	age_mo_int	Age	-0.217	-0.755	0.321	0.426
M	ΔVO2max	age_mo_int	Age:MO	0.049	-0.238	0.336	0.735
M	ΔVO2max	age_mo_int	MO	0.093	-0.44	0.627	0.729
M	ΔVO2max	mo	MO	-0.092	-0.281	0.096	0.332
